# Peanut Genotypes with Reduced Content of Immunogenic Proteins by Breeding, Biotechnology, and Management: Prospects and Challenges

**DOI:** 10.3390/plants14040626

**Published:** 2025-02-19

**Authors:** Tariq Alam, Sachin Rustgi

**Affiliations:** 1 Department of Plant and Environmental Sciences, Clemson University Pee Dee Research and Education Center, Florence, SC 29506, USA; talam@clemson.edu; 2 School of Health Research, Clemson University, Clemson, SC 29634, USA; 3 Center for Human Genetics, Clemson University, Greenwood, SC 29646, USA

**Keywords:** peanut allergy, major allergenic proteins, reduced-immunogenicity genotypes, genetic engineering, genome editing, oral immunotherapy

## Abstract

Peanut allergies affect millions of people worldwide, often causing life-threatening reactions and necessitating strict avoidance. Recent advancements in oral immunotherapy, such as Palforzia™, and IgE-mediated treatments (e.g., Xolair), have improved care options; however, their high costs limit accessibility and widespread utility. To address these challenges, researchers are employing conventional breeding and advanced molecular tools, such as CRISPR editing, to develop peanut lines with reduced levels of major allergenic proteins (Ara h1, Ara h2, Ara h3, and Ara h6). These reduced-immunogenicity genotypes retain their agronomic viability, flavor, and nutritional quality to some extent, offering the potential for cost-effective oral immunotherapy and safe food options for use in public spaces by non-allergic individuals. Rigorous evaluation, including immunological assays and human feeding trials, is essential to confirm their effectiveness in reducing allergic reactions. Adoption will depend on the establishment of clear regulatory guidelines, stakeholder education, and transparent communication of the benefits and risks. With sustained research, public trust, and supportive policies, reduced-immunogenicity peanuts could substantially lower the global burden of peanut allergies. This communication examined the impact of peanut allergies worldwide and explored strategies to develop peanut genotypes with reduced allergen content, including conventional breeding and advanced genetic engineering. It also addressed the challenges associated with these approaches, such as policy and regulatory hurdles, and outlined key requirements for their successful adoption by farmers and consumers.

## 1. Introduction

Peanut (*Arachis hypogaea* L.) is a nutrient-rich legume valued for its high protein (25%), fat (45–50%), and abundance of micronutrients [1]. Although botanically a legume, it is often grouped with tree nuts due to its culinary uses [2,3]. Peanuts are consumed in various forms, including roasted snacks, peanut butter, margarine, cooking oil, and processed foods. They are a vital, cost-effective source of nutrition globally [4]. However, peanuts are among the most potent food allergens, capable of causing severe allergic reactions, including anaphylaxis, in sensitive individuals [5].

Peanut allergy is one of the most common and severe food allergies, recognized by the U.S. Food and Drug Administration as one of nine primary allergens [6]. It affects approximately 6.1 million people in the United States, with cases steadily increasing over the last two decades [7]. Strict avoidance of peanuts and peanut-containing products is essential, but their ubiquity in processed foods makes this challenging [8].

Globally, peanut allergy prevalence is likely underreported, particularly in low- and middle-income countries, due to limited data collection [9,10,11]. In North America, prevalence is well-documented, affecting 2–3% of the population, with rates as high as 5–6% among children [12,13,14]. In contrast, less industrialized regions lack robust healthcare systems and surveillance mechanisms, complicating efforts to gauge the global burden of peanut allergies. Addressing these data gaps through systematic collection and reporting is crucial for public health planning.

In recent years, efforts to manage and treat peanut allergies have progressed beyond simple avoidance strategies. Innovative therapies like Palforzia™ [15,16] and Xolair (Omalizumab) [17] offer new options for patients by reducing allergic reactions. Palforzia™, approved by the FDA in 2020, mitigates the severity of allergic responses in peanut-sensitive individuals through gradual immune system desensitization to small quantities of peanuts. Xolair, an injectable non-selective medication, was recently approved to manage symptoms of food allergies, including peanut allergy [18].

Beyond therapeutic interventions, there is growing interest in biotechnological and breeding strategies to reduce the allergenic properties of peanuts. Advanced genetic engineering techniques aim to develop peanut lines with lower immunogenicity [19]. Genetic engineering encompasses introducing foreign DNA to express or suppress specific genes, as well as genome editing via site-directed mutagenesis. RNA interference (RNAi) has successfully reduced the accumulation of key allergenic proteins in peanuts [20,21]. Similarly, CRISPR-mediated editing of major allergen genes, such as Ara h2, Ara h6, and Ara h7, has shown promising results [22,23]. These modifications aim to lower allergenic protein levels, reducing the risk of severe reactions upon accidental exposure [24]. This could alleviate the need for a strict peanut-free environment in public settings and households. Additionally, these genotypes could facilitate the development of affordable oral therapies to desensitize peanut-sensitive individuals and support the early introduction of peanuts in high-risk infants.

**Figure 1:**
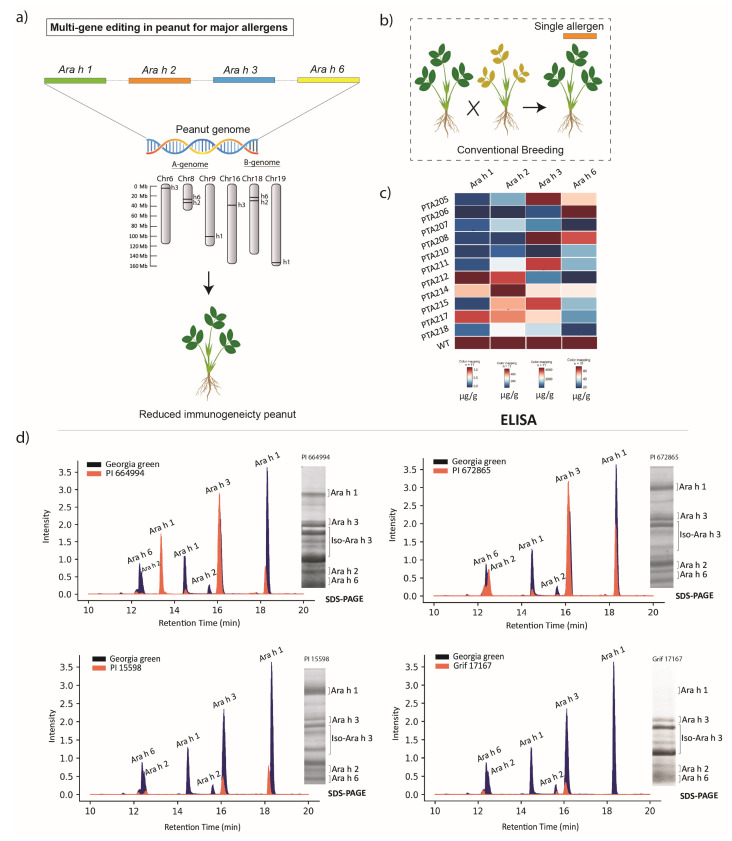
Approaches used for developing reduced-immunogenicity peanut genotypes. (**a**) Targeted editing of major allergen genes (*Ara h1*, *Ara h2*, *Ara h3*, and *Ara h6*) distributed across six peanut chromosomes in three homoeologous groups (see Table S1 for details). (**b**) Selective breeding of peanut genotypes with reduced allergen levels. (**c**) Assessment of allergen content (Ara h1, Ara h2, Ara h3, and Ara h6) in various peanut genotypes. (**d**) Analysis of allergenic protein profiles in different peanut genotypes using LC/MS and SDS-PAGE.

Conventional breeding approaches focus on selecting and crossing peanut genotypes with naturally lower levels of allergenic proteins such as Ara h1, Ara h2, Ara h3, and Ara h6—four major allergens in peanut seeds (Figure 1). Screening efforts utilize germplasm collections, including the U.S. Peanut Mini-core Collection [19,25,26], 27 commercial peanut varieties representing four market classes [27], 151 peanut accessions of the Plant Genetic Resources Institute (PGRI), Pakistan [28], 46 commercial peanut varieties in China [29], and the Peanut Mini-core Collection at the International Crops Research Institute for the Semi-Arid Tropics (ICRISAT) [30].

Screening techniques, such as one- and two-dimensional SDS-PAGE (sodium dodecyl sulfate–polyacrylamide gel electrophoresis) and ELISA (enzyme-linked immunosorbent assay), are commonly used to measure allergen protein levels. These studies have identified genotypes with lower accumulation or a deficiency of major allergenic proteins. However, none of these genotypes were entirely free of all major allergens, a finding that parallels observations in soybean germplasm. In soybeans, gene stacking is employed to combine deficiencies in specific allergenic proteins through genetic crossing [31,32].

These germplasm collections provide the genetic diversity necessary to develop low-allergen peanut genotypes through hybridization and backcrossing, ensuring desirable agronomic traits alongside reduced allergenicity. Wild relatives and landraces further expand the genetic base for breeding programs targeting reduced-immunogenicity peanuts [19].

Genetic engineering efforts in peanuts aim to reduce allergenicity by targeting key allergenic proteins (Ara h1, Ara h2, Ara h3, and Ara h6) using RNAi [20,21] and genome-editing technologies like CRISPR-Cas12a and CRISPR-Cas9 (Figure 1) [22,23,33,34,35]. However, the polyploid complexity of the peanut genome presents challenges, as many genes exist in duplicated forms as homoeologous and paralogous copies. For instance, *Ara h6* is duplicated on chromosome 18, and five additional copies of *Ara h3* are located on chromosome 16 (see Table S1). Despite these complexities, sequence conservation among various copies of a gene makes genome editing feasible.

Recent analyses of diverse peanut lines have revealed a complete absence of some allergenic proteins [19,33,36], potentially due to structural defects in the gene complex or issues with trans-regulators. Additionally, since these genes are not under active selection, many copies may gather mutations, reducing protein accumulation.

The simultaneous targeting of multiple allergen genes can be achieved using methods like LbCas12a, which processes multiple guide RNAs from a single transcript to edit genes located on different chromosomes. Similarly, Cas9 achieves comparable results through specialized gRNA designs, such as incorporating tRNAs to separate individual guide RNAs [22]. Genome editing reagents are delivered through methods like biolistic transformation and viral vectors [22,30,33,36], which enhance editing efficiency. For instance, Biswas et al. [23] demonstrated genome editing in peanut protoplasts; however, protoplast regeneration and reduced allergen production in peanuts were not reported. In contrast, Conner et al. [22] successfully demonstrated inheritable editing of the allergen genes *Ara h2*, *Ara h6*, and *Ara h7* in peanuts.

Public sector research focuses on RNAi, chemical mutagenesis (TILLING), and genome editing to reduce immunogenic proteins (University of Georgia) [22,37]. Research at Texas A&M explores editing allergen genes in peanut protoplasts [20], while Germany’s Institut für Pflanzengenetik focuses on *Ara h1* editing [38]. At NC A&T University, the startup Alrgn Bio detoxifies allergens in blanched peanut seeds using digestive enzymes like trypsin and alcalase [39]. Private sector initiatives, such as MyFloraDNA and Ukko, aim to edit allergenic genes or modify epitopes to reduce allergenicity, using these proteins to desensitize immune responses.

In summary, this review examined the global burden of peanut allergies and strategies to develop reduced-allergenicity peanuts through conventional breeding and advanced genetic engineering. Key challenges include policy and regulatory hurdles, agronomic viability, efficacy in reducing allergic reactions, and consumer acceptance. Addressing these issues can help reduce the public health impact of peanut allergies and facilitate the successful adoption of these innovations.

## 2. Advances in Peanut Allergy Treatment: From Avoidance to Immunotherapy and Beyond

The treatment landscape for peanut allergies has advanced considerably, transitioning from strict avoidance and emergency interventions to proactive desensitization therapies. Historically, individuals with peanut allergies were advised to meticulously avoid peanuts and carry epinephrine auto-injectors to manage accidental exposures [40]. In recent years, oral immunotherapy (OIT) has emerged as a promising treatment option, with its foundation in the initial randomized trial of peanut consumption in infants [41]. In 2020, FDA approved Palforzia™, the first OIT medication for peanut allergies in children aged 4 to 17 [16,42]. More recently, Palforzia™ received approval for use in even younger children (ages 1–3) [43]. This therapy involves the administering of gradually increasing doses of peanut proteins under medical supervision to build tolerance. Other approaches, such as epicutaneous immunotherapy (EPIT) via skin patches and sublingual immunotherapy (SLIT), are also being explored in clinical trials [44]. Major challenges associated with these therapies include their high cost and allergen specificity, as they are only effective against the targeted food allergen. Recently, a wide-spectrum immunoglobulin E (IgE)-mediated therapy called Xolair has been introduced [45]. This treatment is effective against various food allergens, particularly those caused by cross-reactive foods like tree nuts and soybeans. However, while effective, Xolair is expensive and may cause adverse side effects in some cases.

In addition to immunotherapy, efforts have been made to reduce the allergenicity of peanuts through classical breeding [37,46] and genetic modification [20,21,23]. Specifically, different research groups have attempted to develop peanut genotypes with lower levels of allergenic proteins—Ara h 1, Ara h 2, Ara h 3, and Ara h 6—using conventional breeding techniques [19]. Research conducted at Clemson and elsewhere has identified several peanut genotypes with reduced levels of immunogenic proteins. As expected, these screenings revealed that none of the genotypes completely lack all four major allergens. Furthermore, the screening of diploid peanuts suggested that, except for *Arachis diogoi*, all other diploids (*Arachis duranensis*, *A. ipaensis*, *A. cardensii*, and *A. correntina*) generally exhibit a full complement of immunogenic proteins [19].

This initial analysis indicated that domestication has not resulted in an increase in allergen content. Additionally, the study included peanut varieties from six different decades. It showed that, while protein content increased over time due to breeding for higher protein levels, there was no corresponding impact on allergen content (Ingole, Jones, and Rustgi, unpublished data). This pilot study ruled out breeding for increased protein content as the source of the observed rise in peanut allergy incidences. Genetic crossing to stack the missing protein phenotypes of selected lines is currently underway and is likely to yield peanut genotypes with reduced levels of all allergenic proteins.

These conventionally bred reduced-immunogenicity peanuts are expected to increase accessibility by making low-immunogenicity peanut genotypes widely available in public spaces, thereby reducing the risk of accidental exposure to lethal doses of immunogenic proteins and enhancing safety for allergic individuals. Additionally, the widespread availability of hypoallergenic peanuts may help alleviate the financial and logistical burdens associated with maintaining peanut-free environments, ultimately improving the quality of life for individuals with peanut allergies and their families.

Genetic engineering has also been employed to reduce allergenic proteins, with the goal of developing peanut genotypes with lower immunogenicity. Two main approaches have been undertaken. Early efforts focused on silencing one or more genes encoding major allergens using RNA interference, while later efforts involved direct gene editing of immunogenic proteins. In the latter approach, some studies targeted single or multiple sites within a gene of interest, while others focused on base editing specific epitopes to diminish or eliminate IgE reactivity. Gene-editing strategies targeting one or more sites within a gene have led to various outcomes, including small insertions/deletions (InDels), substitutions, or large deletions, depending on the number of targets per gene, ultimately resulting in reduced accumulation or complete elimination of allergenic proteins. In the following paragraphs, we will further explore the multi-gene editing strategies employed by our team and others.

Since peanut allergy has been recognized as a significant health concern, and even a small dose of the allergen (24 parts per million) can trigger an immune reaction, a multifaceted approach—combining avoidance, emergency preparedness, immunotherapy, and scientific innovation—continues to evolve to improve patient outcomes.

## 3. Peanut-Processing Technologies for Allergen Reduction

Post-harvest peanut processing using thermal and non-thermal methods has been shown to reduce allergenicity. Thermal treatments, such as boiling, roasting, and autoclaving, can alter protein structures, potentially decreasing or increasing IgE-binding capacities. For instance, boiling peanuts for extended periods has been shown to reduce the allergenic potential of major proteins like Ara h 1, Ara h 2, and Ara h 3 [47]. On the other hand, roasting peanuts has been found to have an adverse effect, as it is suspected to expose the IgE-binding sites in the protein [48]. Non-thermal approaches, including enzymatic hydrolysis and fermentation, have also demonstrated effectiveness in modifying allergenic proteins, thereby reducing their reactivity. These processing techniques offer promising avenues for producing hypoallergenic peanut products, enhancing safety for allergic individuals [49]. Novel processing technologies, such as High-Pressure Processing (HPP) and Pulsed Ultraviolet Light (PUV), have shown substantial decreases in allergenicity by altering protein structures and reducing solubility [50,51]. Furthermore, hurdle technology, which integrates multiple processing methods—such as combining HPP with heat or ultrasonication with enzymatic hydrolysis—has proven to enhance the effectiveness of mitigating peanut allergenicity [50,52,53]. Together, these advancements underscore the potential of both integrated and innovative processing strategies to effectively reduce the risks associated with peanut allergy.

## 4. Simultaneous Editing of Multiple Allergens

Developing peanut genotypes with reduced immunogenicity involves simultaneously reducing multiple allergenic proteins, notably Ara h 1, Ara h 2, Ara h 3, and Ara h 6. Genetic engineering techniques, such as CRISPR and RNA interference, enable the efficient reduction of these allergens in a single step. These methods are more efficient than conventional breeding, which requires extensive genetic crossing and selection over multiple generations to effectively pyramid the desired knockout or knockdown alleles for all major allergens in a homozygous genetic background. Our preliminary results, based on protein markers, demonstrated that it is possible to pyramid deficiencies in multiple groups of allergenic proteins within a single genotype. This approach significantly lowers the risk of severe allergic reactions while preserving desirable traits such as total protein content, flavor, nutritional attributes, and agronomic performance (Jones, Ingole, Rustgi, unpublished data). Field performance studies on transgenic lines deficient in more than one major allergen support this possibility. Furthermore, Chu et al. [54] demonstrated that Ara h 2 and Ara h 6 deficiencies do not increase plant susceptibility to *Aspergillus* species. Similarly, the simultaneous silencing of Ara h 1, Ara h 2, and Ara h 3 was shown to have no adverse impact on flavor [20]. Additionally, enzyme treatments applied to seeds during pre-processing also did not affect flavor [55]. These early studies, along with our observations, confirm that it is feasible to develop peanuts with reduced allergen content without compromising agronomic performance or quality.

## 5. Policy and Regulatory Considerations in Developing Reduced-Immunogenicity Peanuts

Experience with transgenic crops like maize, cotton, soybean, papaya, and eggplant demonstrates that genetically engineered crops can be environmentally sustainable and safe for human use [56]. The global scientific consensus supports the safety of such crops, which may also extend to genetically modified peanut lines including those designed to reduce allergenicity.

## 6. Perspectives in Reducing Peanut Allergenicity

The development of reduced-immunogenicity peanut genotypes through conventional breeding and genetic engineering presents a promising strategy to mitigate the significant risk of severe reactions from unintentional peanut exposure and its life-threatening outcomes, as well as to address the global need for “peanut-free” environments. Additionally, these efforts may pave the way for affordable peanut desensitization therapies. The modified peanuts aim to lower allergenic protein content, thereby reducing the risk of severe allergic reactions upon accidental exposure [24]. While conventional breeding methods have shown initial promise, genetically engineered mutants offer a faster pathway to achieving significant reductions in allergenicity. The first approach provides an immediately implementable solution, as it relies on widely accepted breeding procedures [57]. In contrast, the genetic engineering approach, even if approved by regulators in certain parts of the world, still faces public resistance but may become more commonplace in the future [57]. Both strategies, however, face ongoing challenges, including the need to establish a contamination-free supply chain from farm to fork, encompassing production, shelling, and processing (especially for processed products).

Major seed storage proteins, including Ara h 1, Ara h 2, Ara h 3, and Ara h 6, which constitute 45–48% of seed protein content, are primary triggers of allergic reactions in susceptible individuals [58]. Genetic engineering technologies, such as CRISPR and RNA interference, have enabled the precise modification of these proteins in peanut seeds [24]. By simultaneously targeting multiple allergenic proteins, researchers aim to develop peanut lines with significantly lower allergen content [33]. This approach not only reduces the severity of allergic reactions upon accidental exposure but also holds promise for creating affordable genotypes with reduced allergenic protein levels. These could serve as oral immunotherapy by containing just enough allergenic proteins to train and desensitize the immune system. Moreover, studies on transgenic peanut lines with reduced immunogen content suggest that genotypes with reduced allergen content are likely to retain desirable traits, such as flavor, nutritional value, and productivity.

## 7. Key Requirements for Developing Reduced-Immunogenicity Peanut Genotypes

To successfully develop and utilize peanut genotypes with reduced immunogenicity, three essential requirements must be met. Additionally, key considerations for breeders, extension specialists, producers and consumers are outlined separately below.

1. Agronomic Viability and End-use Quality: The modified peanut lines must maintain high and stable crop yields without adding to the production costs. Furthermore, in view of climate change it is important to study the impact of heat, drought, and management practices (nitrogen, sulfur fertilization, conservation tillage) on the heritability of the reduced-allergen trait. Furthermore, it is vital to maintain/enhance the nutritional quality of the lines, specifically total protein content, as reduction in protein content would make it challenging to get FDA approvals for the release of such a material. This ensures the developed genotypes remain relevant for farmers and consumers by performing well in the field without compromising nutritional value, flavor, or other valuable properties.
2. Demonstrated Efficacy: The effectiveness in reducing allergic reactions must be proven using a stepwise approach, which includes biochemical and immunological testing, including the enzyme-linked immunosorbent assays (ELISA), with the antibodies raised against specific allergens and IgE from the sera of peanut allergic individuals, and basophil activation test (BAT) with the cells derived from allergic individuals followed by double blind human feeding trials. Sensitive individuals should exhibit reduced or no allergic responses upon exposure. Additionally, the low allergen content must remain stable during storage, processing, and cooking to ensure safety and quality throughout the food supply chain.
3. Adoption by Stakeholders: Widespread adoption by both farmers and consumers is essential. This requires proper incentives, education—including guidance on maintaining line purity during production and preventing contamination during shelling and processing—and effective delivery strategies. These measures will ensure that safer peanut genotypes are accessible to individuals with allergies, while also encouraging farmers to grow them, shellers to handle them properly, and processors to incorporate them into various products.

Genetic engineering should go hand in hand with conventional breeding, as it may not be accepted today but will be a mainstream approach in plant breeding tomorrow. In this context, the following specific actions and policy interventions would be needed:

1. Expand Germplasm Collections: There is a need to broaden peanut germplasm collection and screen existing germplasm to better characterize the natural diversity of allergenic proteins. This will provide genotypes suitable for conventional breeding approaches and genetic material to enhance understanding of genetics of allergen accumulation in peanut seeds.
2. Genotype and Tissue-culture Independent Biomolecule Delivery Methods. It will allow an unbiased and accelerated delivery of the genome-editing/base-editing reagents to the genotypes of interest without any challenge. It may also include the development of engineered *Agrobacterium* strains that will serve that purpose or identification of environmentally safe nanomaterial that would allow safe delivery of these reagents to the genotypes of choice in no time.
3. Ensure Accessibility and Affordability: Making reduced-immunogenicity peanut genotypes accessible and affordable for vulnerable populations is essential. Their deployment should be backed by rigorous clinical trials that confirm their effectiveness in minimizing allergic responses.
4. Provide Independent Safety Information: Despite broad scientific endorsement of the safety of genetically engineered crops by over 100 Nobel laureates and scientific academies worldwide, there is a need for wide surveys to understand and inform public perspective to provide independent, science-based information on the safety of genetically modified peanuts to facilitate decision making. Transparent communication can address public concerns and foster trust in these innovations.
5. Update Regulatory Frameworks: Many countries lack appropriate regulatory frameworks or have restrictive policies. Local non-profit organizations can play a key role in educating the public, media, and policymakers about the health and socio-economic implications of introducing reduced-immunogenicity peanuts. Publicly funded organizations, supported by charitable entities and coordinated by governments alongside local academics, could provide a non-profit avenue to reach populations in need.

By addressing these areas, the full potential of reduced-immunogenicity peanut genotypes can be realized, providing a groundbreaking solution to a significant public health challenge. Collaboration among scientists, policymakers, farmers, and consumers will be essential to advancing these innovations from the laboratory to the field and, ultimately, to the tables of those who need them most.

## Data Availability

The data reviewed here is available in the manuscript.

## Supplementary Materials

The following supporting information can be downloaded at: https://www.mdpi.com/article/10.3390/plants14040626/s1, Table S1: Genomic locations of the major peanut allergen genes *Ara h1*, *Ara h2*, *Ara h3*, and *Ara h6*, distributed across six chromosomes in three homoeologous groups.
